# Millimeter-Wave Multi-Channel Backscatter Communication and Ranging with an FMCW Radar

**DOI:** 10.3390/s22197104

**Published:** 2022-09-20

**Authors:** Sining An, Xiangyuan Bu, Henk Wymeersch, Herbert Zirath, Zhongxia Simon He

**Affiliations:** 1 Microwave Electronics Laboratory, Department of Microtechnology and Nanoscience, Chalmers University of Technology, SE-41296 Gothenburg, Sweden; 2 School of Information Science and Electronics, Beijing Institute of Technology, Beijing 100081, China; 3 Department of Electrical Engineering, Chalmers University of Technology, SE-41296 Gothenburg, Sweden; 4 SinoWave AB, SE-43650 Hovås, Sweden

**Keywords:** active reflector, backscatter communication, FMCW, millimeter-wave, multi-channel, multi-tag, radar, radar communication, RF tag, spread spectrum, uplink

## Abstract

A multi-channel backscatter communication and radar sensing system is proposed and demonstrated in this paper. Frequency modulated continuous wave (FMCW) radar ranging is integrated with simultaneous uplink data transmission from a self-packaged active radio frequency (RF) tag. A novel package solution is proposed for the RF tag. With the proposed package, the RF tag can transmit a 32-QAM signal up to 2.5 Gbps and QPSK signal up to 8 Gbps. For a multi-tag scenario, we proposed using spread spectrum code to separate the data from each tag. In this case, tags can be placed at arbitrary locations without adjacent channel interference. Proof-of-concept simulations and measurements are demonstrated. A 625 Mbps data rate is achieved in a dual-tag scenario for two tags.

## 1. Introduction

Backscatter communication has the advantage of low power consumption for wireless telemetry uplink communication. Over the past few decades, point-to-point backscatter communication has been widely deployed in the application of radio frequency identification (RFID) for a passive RFID tag to report an ID to an enquiring reader over the near field (typically several tens of centimeters) [1,2,3,4,5]. Backscatter communications are widely used with low data rates or low modulation order signals, due to the simplified passive tag in use [6,7,8,9,10,11,12,13]. Table 1 summarizes some of the recently published backscatter communication system performances.

In the traditional backscatter communication system, the carrier signal is a single-tone continuous wave. In this case, the transmitted carrier signal and received modulated signal are synchronized with no offset frequency. There is only a phase offset that exists between those two signals, which is easy to remove. Besides single tone signals, linear frequency modulated signals, such as FMCW, can also be used as the carrier. In this case, there is a frequency offset remaining in the received signal. As an extra step of estimating this frequency, offset needs to be taken. This offset frequency gives extra information about the distance to the tag. The backscatter communication system coexists with the FMCW radar system under this situation. We can call it an FMCW radar-communication system.

The FMCW radar-communication system has been studied [12,13,14,15]. In paper [12], a switch is used in the tag to modulate the on-off keying (OOK) signal to the FMCW. It demonstrates the range estimation capability of such systems. Paper [13] simulates the capabilities of simultaneous localization and data transmission of the FMCW radar-communication system. In paper [14], the OOK modulation scheme is also used for communication. In addition, it demonstrates that the FMCW ranging approach can be integrated with a simultaneous data transmission with a data rate of 37.5 Mbps in lab measurements. Paper [15] demonstrates the FMCW radar-communication system in a real working scenario of car-to-car communication at 24 GHz.

However, few papers [16] dig into the performance of the FMCW radar-communication system with multiple tags. The separation between tags is a challenge in this scenario. Paper [16] presented proof-of-principle measurements using a brass-board S-band (2.45 GHz) radar with 40-MHz bandwidth, showing simultaneous ranging and demodulation of two tags at ranges of 15 and 33 m in a cluttered indoor environment. This paper studied the multi-tag scenario, and in order to separate information from two tags, tags should be sufficiently physically separated so that two signals at the receiver side will not overlap in the spectrum. When the symbol rate increases, the minimum spacing needs to increase accordingly to avoid the spectrum overlapping. Otherwise, the FMCW chirp signal needs to have larger bandwidth or a shorter sweep time. Both cases require either a hardware update or infrastructure re-installation, which complicates the implementation. Besides the described space division multiple access methods, there are also other solutions to avoid collisions between multiple tags; for example, frequency/code/time division multiple access (FDMA/CDMA/TDMA) [17,18]. CDMA is a suitable candidate because it does not require a radar hardware update. The multi-tag backscatter communication with the FMCW radar system can be really useful in certain application scenarios. In the next section, we will introduce a smart traffic scenario that could benefit from this system.

**Table 1:** Recently reported backscatter communications.

Ref.	Single Tag Data Rate	Modulation	Dual-Tag Data Rate	RF Signal Type	RF Freq. (GHz)	Radar Sensing Ability
[6]	1 kbps	AM	--	CW	60	no
[7]	1 kbps	OOK/FSK	--	CW	0.867	no
[8]	336 kbps	Square wave	--	CW	77	no
[9]	400 kbps	QPSK	--	CW	0.915	no
[10]	96 Mbps	16-QAM	--	CW	0.915	no
[11]	2.5 Mbps	32-QAM	--	CW	5.8	no
[14]	37.5 Mbps	OOK	--	FMCW	34.3–34.8	yes
[16]	--	BPSK	10 kbps (BPSK)	FMCW	2.43–2.47	yes
This	2.5 Gbps/ 8 Gbps	32-QAM/ QPSK	625 Mbps (BPSK)	FMCW	75.2–78.2	yes

## 2. Smart Traffic Infrastructure

**Figure 1:**
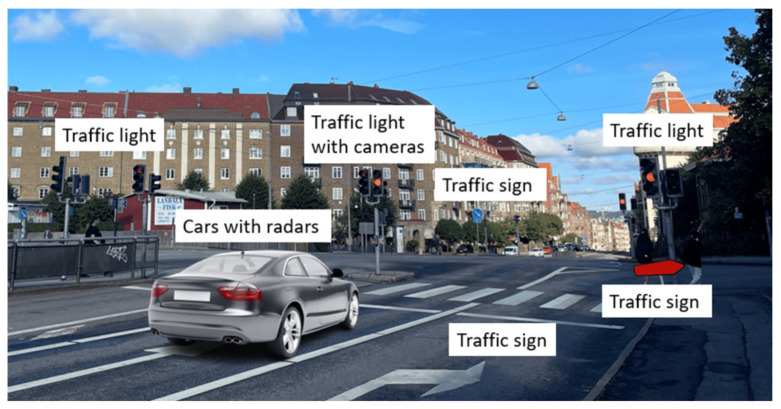
An illustration of the scenario of interest.

We consider a smart traffic system where the traffic infrastructure is equipped with passive tags, and vehicles are equipped with automotive radars. An intersection with traffic lights is a potential application scenario, as Figure 1 shows.

Cameras attached to traffic lights are widely installed nowadays. Pictures and videos taken by cameras are currently mostly used for monitoring and can only be retrieved by the relevant government departments. With a radar-communication system, important traffic news, such as accident reports or temporary road obstacle locations, can be transmitted wirelessly directly to vehicles. In that case, drivers can re-plan their route in advance and avoid traffic congestion. Moreover, under extreme weather conditions, such as heavy rain and snow, the traffic signs and traffic lights are difficult to see. If the traffic information can be transmitted to vehicles wirelessly and displayed locally to the driver, fewer accidents occur. For these purposes, we require an information broadcast system. A backscatter communication system is suitable for this application. Traffic signs and lights carry certain information, which is perfect to use a tag to broadcast. In an intersection scenario, there are normally multiple signs, traffic lights, and cameras installed, so multiple tags are needed.

Radars are prevalent in modern vehicles and are used to detect the surrounding obstacles, road users, or other vehicles. FMCW radars are the most commonly used vehicle radars for their ability to detect both the position and Doppler velocity of the surrounding target [19,20]. If infrastructures are equipped with tags and vehicles have the FMCW radar installed, communication and radar sensing can be realized simultaneously. The FMCW radar chips can be reused in this radar-communication system. Only the signal processing requires an update.

In this paper, a multi-channel backscatter communication with an FMCW radar system is presented. A novel package solution for the tag is proposed. The radar-communication system is tested with commercial E-band frontend modules. The single tag measurement demonstrates the highest transmission data rate of 8 Gbps and the highest modulation order signal of 32-QAM which shows the remarkable performance of the self-packaged tag. The frequency offset between the transmitter and the receiver is estimated for range measurement. In multi-tag measurement, two tags are used in the proposed radar-communication system. 625 Mbps BPSK signals are successfully transmitted with both tags. Table 1 compares the backscatter communication performance of related works.

The paper is organized as follows. In Section 3, the principle of backscatter communication with FMCW is presented. Then, the multi-tag message separation is introduced in Section 4. In Section 5, the signal processing framework of the proposed system is introduced. The novel package solution for the tag is presented in Section 6, and its performance test results are also given in this section. The system measurement of the proposed system is presented in Section 7. Finally, the conclusion and discussion are given in Section 8.

## 3. Backscatter Communication with FMCW

**Figure 2:**
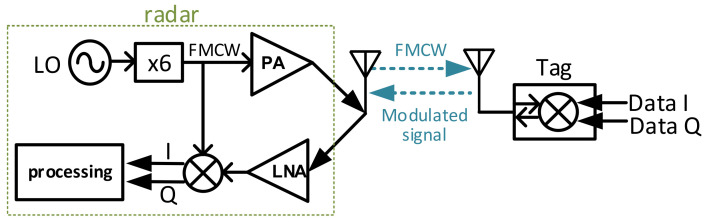
The backscatter communication system with FMCW radar structure.

An overview of the proposed backscatter communication system with FMCW radar structure is shown in Figure 2.

On the radar (vehicle) side, a local oscillator (LO) generates a low-frequency linear frequency modulated (LMF) signal. Then, the LMF signal is multiplied by a frequency multiplier to millimeter-wave frequency. An FMCW signal is generated, for which a single chirp can be represented as:

(1) $$x\left(t\right)=e^{j\left(2\pi f_{0}t+\pi \frac{B}{T}t^{2}+\varphi_{0}\right)}$$

where *B* is the chirp bandwidth, *T* is the chirp duration, $f_{0}$ is the initial frequency of the chirp and $\varphi_{0}$ is the initial phase. The FMCW signal is amplified and then sent out by an antenna. At the tag (infrastructure) side, the received FMCW signal is modulated with data and sent back to the radar. The data is modulated by mixing the FMCW with the baseband signal as

(2) $$y\left(t\right)=x\left(t\right)\times \sum A\left(n\right)e^{j\theta \left(n\right)}g\left(t-nT_{s}\right)$$

where $g\left(t\right)=\left\{\begin{array}{c} 1,\;\left|t\right|<T_{s}/2\\ 0,\;\left|t\right|>T_{s}/2 \end{array}\right.$, $T_{s}$ is the symbol period. When the signal is received by the radar, it becomes

(3) $$s\left(t\right)=H\times y\left(t-\Delta t\right)+n\left(t\right)$$

where $n\left(t\right)$ is the noise, H is the attenuation due to path loss, Δt is the time delay between the transmitted and the received signal at the radar side. After mixing with the local FMCW, we have

(4) $$r\left(t\right)=H\times y\left(t-\Delta t\right)\times e^{-j\left(2\pi f_{0}t+\pi \frac{B}{T}t^{2}+\varphi_{0}\right)}+n^{\prime }\left(t\right)\;=He^{j\left[2\pi f_{0}\left(t-\Delta t\right)+\pi \frac{B}{T}{\left(t-\Delta t\right)}^{2}+\varphi_{0}\right]}\times \sum A\left(n-\Delta n\right)e^{j\theta \left(n-\Delta n\right))}g\left(t-\Delta t-nT_{s}\right)\times e^{-j\left(2\pi f_{0}t+\pi \frac{B}{T}t^{2}+\varphi_{0}\right)}+n^{\prime }\left(t\right)\;=He^{j\left(-2\pi f_{0}\Delta t+\pi \frac{B}{T}\Delta t^{2}-2\pi \frac{B}{T}\Delta t\cdot t\right)}\times \sum A\left(n-\Delta n\right)e^{j\theta \left(n-\Delta n\right)}g\left(t-\Delta t-nT_{s}\right)+n^{\prime }\left(t\right)$$

where $-2\pi f_{0}\Delta t+\pi \frac{B}{T}\Delta t^{2}$ is a fixed phase, $-2\pi \frac{B}{T}\Delta t\cdot t$ corresponds to a fixed frequency component of $\frac{B}{T}\Delta t$. $\sum A\left(n-\Delta n\right)e^{j\theta \left(n-\Delta n\right)}g\left(t-\Delta t-nT_{s}\right)$ is the received symbol from the tag. Here, $\Delta n=\lfloor \frac{\Delta t}{Ts}\rfloor$, Δt=2Δd/c, where Δd is the range between the radar and the tag, and c is the speed of light. Equation (4) can be rewritten as:

(5) $$r\left(t\right)=He^{j\left(\varphi^{\prime }-2\pi \Delta f\cdot t\right)}\times \sum A\left(n-\Delta n\right)e^{j\theta \left(n-\Delta n\right)}g\left(t-\Delta t-nT_{s}\right)+n^{\prime }\left(t\right)$$

where $\varphi^{\prime }=-2\pi f_{0}\Delta t+\pi \frac{B}{T}\Delta t^{2}$, $\Delta f=\frac{B}{T}\Delta t$. By analyzing the offset frequency Δf from the received signal $r\left(t\right)$, the range information can be estimated. Then after the removal of the offset frequency, the data can be recovered. The offset frequency is related to chirp bandwidth *B*, duration *T*, and the range between the radar and the tag. With wider bandwidth and shorter duration, the offset frequency is larger.

Besides the signal reflected by the tag, there is also a radar signal reflected by near objects. The radar signal reflected by other objects can be represented as

(6) $$c\left(t\right)=\overset{K}{\underset{k=1}{\sum }}H_{k}x\left(t-\Delta t_{k}\right)$$

where $H_{k}$ and $\Delta t_{k}$ are the attenuation and the travel time delay of the *k*-th object. When $c\left(t\right)$ mixed with the local FMCW signal, it becomes

(7) $$r_{c}\left(t\right)=\overset{K}{\underset{k=1}{\sum }}H_{k}e^{j\left(\varphi_{k}-2\pi \Delta f_{k}\cdot t\right)}$$

This is a traditionally received signal of an FMCW radar. The range between radar and objects can be estimated from $\Delta f_{k}$.

The total received signal is

(8) $$r\left(t\right)=He^{j\left(\varphi^{\prime }-2\pi \Delta f\cdot t\right)}\times \sum A\left(n-\Delta n\right)e^{j\theta \left(n-\Delta n\right)}g\left(t-\Delta t-nT_{s}\right)+n^{\prime }\left(t\right)+\overset{K}{\underset{k=1}{\sum }}H_{k}e^{j\left(\varphi_{k}-2\pi \Delta f_{k}\cdot t\right)}$$

Compared with the traditional backscatter communication system, the difference is the carrier signal. The FMCW signal is used instead of a single-tone CW signal. This result in a frequency offset Δf in $r\left(t\right)$. When there is no tag in the view, the proposed system can work normally as an FMCW radar.

To avoid unintentional jamming between the FMCW radar signals and communication signals from tags, as well as distinguish signals from different tags, signature codes are used. This will be discussed in the next section.

## 4. Multi-Tag Scenario

**Figure 3:**
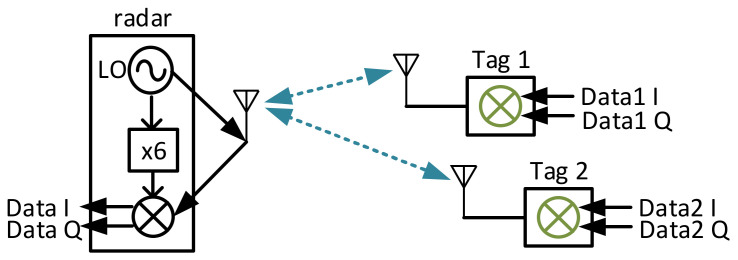
The proposed backscatter communication system with FMCW radar in a multi-tag scenario.

An overview of the proposed backscatter communication system with FMCW radar structure in a multi-tag scenario is shown in Figure 3.

When there are multiple tags in the view, each tag modulates individual data to the FMCW signal and sends it back to the radar side. The received signal under this scenario can be represented as

(9) $$s\left(t\right)=\overset{I}{\underset{i=1}{\sum }}H_{i}\times y_{i}\left(t-\Delta t_{i}\right)+n\left(t\right)+c\left(t\right)$$

where $c\left(t\right)$ represents the reflected signal from other objects and $n\left(t\right)$ is the noise. When it mixes with the local FMCW, it becomes

(10) $$r\left(t\right)=\sum_{i=1}^{I}H_{i}e^{j\left(\varphi_{i}{}^{\prime }-2\pi \Delta f_{i}t\right)}\times \sum A_{i}\left(n-\Delta n_{i}\right)e^{j\theta_{i}\left(n-\Delta n_{i}\right)}g\left(t-\Delta t_{i}-nT_{s}\right)+n^{\prime }\left(t\right)+\sum_{k=1}^{K}H_{k}e^{j\left(\varphi_{k}-2\pi \Delta f_{k}\cdot t\right)}$$

**Figure 4:**
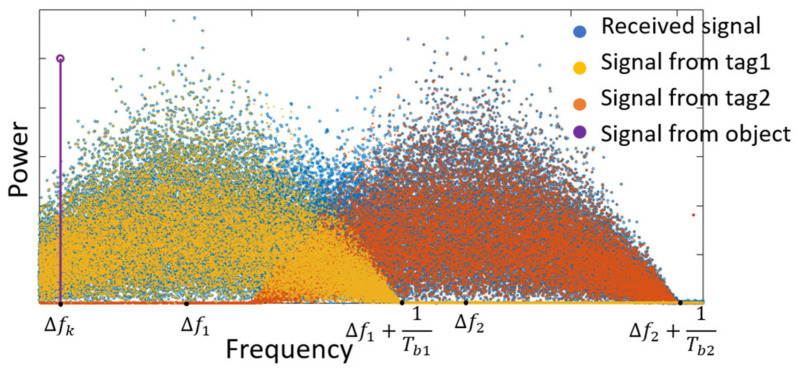
The spectrum of a down-converted received signal from two tags.

Equation (10) shows that reflected signals from tags and objects have different offset frequency $\Delta f_{i}$ and $\Delta f_{k}$. Assuming there are two tags and one object in the view, their spectrum can be shown in Figure 4.

The spectrum of the received signal from the object can be represented as the purple line. There is only a single frequency tone that represents $\Delta f_{k}$, which can be selected by a bandpass filter (BPF) when its spectral power density is larger than the signal from tags. The spectrum of received signals from two tags are represented as yellow and orange dots. They have offset frequencies of $\Delta f_{1}$ and $\Delta f_{2}$, respectively. Their symbol rates are $1/T_{b1}$ and $1/T_{b2}$. When the symbol rate is large and the difference between two frequency offsets $\Delta f_{2}-\Delta f_{1}$ is small, the spectrum of two signals will overlap, as Figure 4 shows. The spectrum of the total signal received from the two tags can be represented by the blue dots. Two signals cannot be separated from their spectrum.

Paper [16] studied the requirement of the distance separation of tags without spectrum overlap, which is

(11) $$\frac{B}{T_{p}}\frac{2\Delta r_{min}}{c}\geq \left[\frac{1}{T_{b1}}+\frac{1}{T_{b2}}\right]$$

where $1/T_{b1}$ and $1/T_{b2}$ are the symbol rate of two transmitted signals from two tags, $T_{p}$ is the FMCW chirp duration, *B* is the chirp bandwidth, *c* is the speed of light, and $\Delta r_{min}$ is the minimum separation range. In this case, the symbol rate of transmitted signals is limited by the distance and FMCW chirp. If the FMCW radar has a chirp bandwidth of 1 GHz, a duration of 1 ms, and the distance between the two tags is 30 m. In this case, the total symbol rate of two tags needs smaller than

(12) $$\frac{10^{9}\times 2\times 30}{10^{-3}\times 3\times 10^{8}}=2\times 10^{5}=200\;\mathrm{Kbaud}$$

By using a more advanced FMCW radar or placing two tags further apart from each other, the system will be able to transmit the signal with a higher symbol rate. A commercial FMCW radar normally has a chirp bandwidth smaller than 1 GHz, and a duration time longer than 1 ms. For the smart traffic scenario introduced in Section 2, the distance between two tags is normally a few dozen meters. In this case, it is difficult to further increase the communication symbol rate up to megabaud.

In this paper, we propose to use CDMA with backscatter communication and radar sensing system to avoid collisions between multi-tag and jamming between signals from tags and objects. For each tag, an individual signature code is used to modulate the original data at the baseband. By using the signature code to represent the symbol, the spectrum of the baseband signal is spread, and its spectral power density is decreased accordingly. On the radar side, the received signal will correlate to each signature code to recover the data of each tag.

Assume the signature code chip $d\left(n\right)\;$ has a length of *L* bits. When the signature code correlates with itself,

(13) $$\overset{L}{\underset{n=0}{\sum }}d\left(n\right)\times d\left(n\right)=L,\;\;d\left(n\right)=\left\{-1,1\right\}.$$

The power of *L* bits will be summed up. When the signature code correlates with other signature codes,

(14) $$\overset{L}{\underset{n=0}{\sum }}d_{1}\left(n\right)\times d_{2}\left(n\right)\approx 0<L,\;\;d_{1}\left(n\right),d_{2}\left(n\right)=\left\{-1,1\right\}.$$

When the signature code correlates with other radar reflection signals from an object, the correlation result can be represented as

(15) $$\int_{t=0}^{LT_{s}}d\left(t\right)\times e^{j\left(\varphi_{k}-2\pi \Delta f_{k}\cdot t\right)}dt$$

where $T_{s}$ is the symbol period. If the symbol rate of the signature code $\frac{1}{T_{s}}$ is much larger than $\Delta f_{k}$, then $e^{j\left(\varphi_{k}-2\pi \Delta f_{k}\Delta t\right)}$ doesn’t change too much within $LT_{s}$ time. When $\sum_{n=0}^{L}d\left(n\right)=0$ holds, then

(16) $$\int_{t=0}^{LT_{s}}d\left(t\right)\times e^{j\left(\varphi_{k}-2\pi \Delta f_{k}\cdot t\right)}dt\approx 0$$

This signal is removed by correlation. In this case, the correlation increases the signal-to-interference ratio (SIR) and signal-to-noise ratio (SNR) of the target tag signal. Hence, data from each tag can be distinguished.

As mentioned before, the normal radar signal from other objects appears as a single tone on the spectrum at the receiver. To maintain the normal FMCW radar sensing functionality, the single tone needs to be selected by a BPF. This requires its SIR and is high enough within the BPF’s bandwidth. From the radar signals perspective, the signals from tags are interferences. To lower the interference power, the tags in use are normally passive. Furthermore, benefitting from the signature codes, the power of the signals from tags can be re-enforced at the receiver side by applying correlation, which means that the tag does not need to provide a large, transmitted power. The idea is similar to the spread spectrum communication. In this case, the normal radar signal can guarantee its SIR to realize sensing while the data from tags can also be recovered. Alternatively, the communication signal can be reconstructed after data recovery and subtracted from the observation (10), prior to applying standard signal processing for sensing.

The main limitation of the communication data rate is the bandwidth of the radar module and the tag in use. The received signal $s\left(t\right)$, which is also the transmitted signal from the tag, has a bandwidth of $B+2B_{s}$, where B is the FMCW chirp bandwidth and $B_{s}=\frac{1}{T_{s}}$ is the baseband signal bandwidth. $B+2B_{s}$ need to be smaller than the RF bandwidth of the radar module and the tag. After mixing with the local FMCW signal, $r\left(t\right)$ has a bandwidth of $B_{s}+\Delta f_{k}$. It needs to be smaller than the baseband bandwidth of the radar module.

## 5. Signal Processing Framework

### 5.1. Single Tag Scenario and Range Estimation

When there is only one tag in the view, the received signal can be represented as (5) after mixing with the local FMCW signal. There is only a fixed frequency offset and a phase offset left. By using fast Fourier transformation (FFT) and a phase-locked loop (PLL), we can derive the offset frequency and offset phase. A pilot signal of long zeros can be placed ahead of the data to assist in frequency offset extraction. The distance between the radar and the tag can be calculated by

(17) $$\Delta d=\frac{\Delta f\times T\times c}{2B}$$

### 5.2. Multi-Tag Separation and Communication Processing

**Figure 5:**
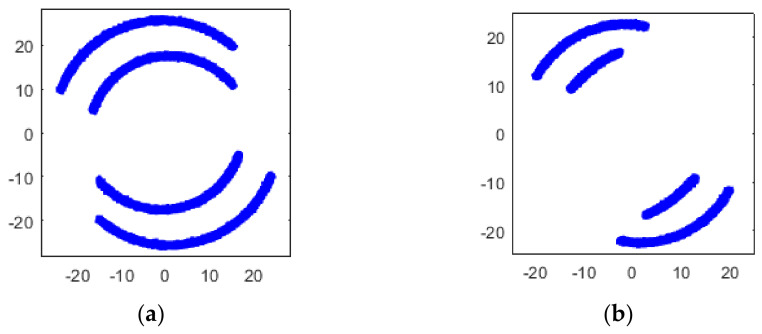
Simulated constellation diagrams of (**a**) $RX_{1}$ and (**b**) $RX_{2}$ with $\Delta f_{1}$ of 500 Hz and $\Delta f_{2}$ of 250 Hz.

When there are multiple tags and objects in the view, each tag is assigned an individual signature code. The received down-converted signal can be represented as (10). The down-converted signal $r\left(t\right)$ needs to first be correlated with each signature code to increase the SNR and SIR. The distance between tags and the radar is random, and the time delay between signals from different tags is also random. In this case, the correlation between two different signature codes is represented as

(18) $$\int_{t=0}^{LT_{sym}}d_{1}\left(t\right)\times d_{2}\left(t-\Delta t\right)dt=f\left(\Delta t\right)\ll L$$

where Δt≠0 is the time delay difference between two signals from two tags. After the correlation, there is still a little power of the signal from another tag left. Assume there are two tags. $TX_{1}$ and $TX_{2}$ are the signals from two tags. $RX_{1}$ and $RX_{2}$ are two correlation results on the radar,

(19a) $$RX_{1}=a_{1}TX_{1}\times e^{j\left(\theta_{1}+\Delta \omega_{1}t\right)}+b_{1}TX_{2}\times e^{j\left(\theta_{2}+\Delta \omega_{2}t\right)}$$

(19b) $$RX_{2}=a_{2}TX_{1}\times e^{j\left(\theta_{1}+\Delta \omega_{1}t\right)}+b_{2}TX_{2}\times e^{j\left(\theta_{2}+\Delta \omega_{2}t\right)}.$$

where $a_{1}\gg b_{1}$, $a_{2}\ll b_{2}$, which means in $RX_{1}$, $TX_{1}$ has the majority of the power, while in $RX_{2}$, $TX_{2}$ has the majority of the power, as Figure 5 shows. 

As a result, the offset frequency $\Delta \omega_{1}$ and phase offset $\theta_{1}$ can be estimated from $RX_{1}.$ Similarly, $\Delta \omega_{2}$ and $\theta_{2}$ can be estimated from $RX_{2}$. The offset frequency estimation has two steps. First, apply FFT to $RX_{1}$ and $RX_{2}$ to get a coarse frequency estimation. Second, a phase-locked loop is applied to further track the frequency and phase offset.

After removing the frequency offset from $RX_{1}$ and $RX_{2}$, they become

(20a) $$RX_{1}{}^{\prime }=RX_{1}\times e^{-j\left(\theta_{1}+\Delta \omega_{1}t\right)}=a_{1}TX_{1}+b_{1}TX_{2}e^{j\left[\theta_{2}-\theta_{1}+(\Delta \omega_{2}-\Delta \omega_{1}\right)t]}$$

(20b) $$RX_{2}{}^{\prime }=RX_{2}\times e^{-j\left(\theta_{2}+\Delta \omega_{2}t\right)}=a_{2}TX_{1}e^{-j\left[\theta_{2}-\theta_{1}+(\Delta \omega_{2}-\Delta \omega_{1}\right)t]}+b_{2}TX_{2}$$

**Figure 6:**
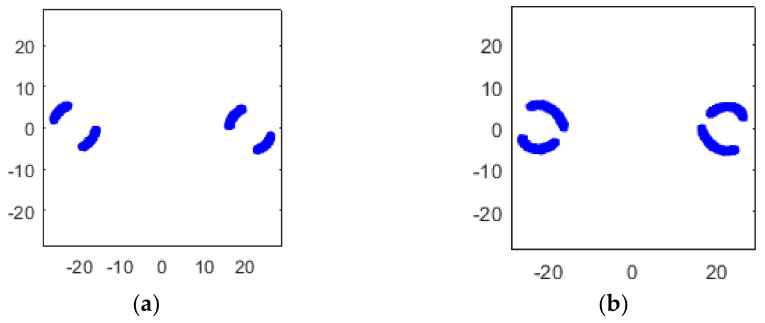
Simulated constellation diagrams of $RX_{1}{}^{\prime }$ with $\Delta f_{2}-\Delta f_{1}$ of (**a**) 250 Hz and (**b**) 500 Hz.

Simulated constellation diagrams of $RX_{1}{}^{\prime }$ with different $\Delta \omega_{2}-\Delta \omega_{1}$ are shown in Figure 6.

### 5.3. Equalization

To further minimize the error vector magnitude (EVM), we need to make the constellation points as concentrated as possible. This can be done via blind equalization.

To concentrate the constellation points, the interference signal from another tag needs to be removed. The $TX_{2}$ part needs to be removed from $RX_{1}{}^{\prime }$, and the $TX_{1}$ part needs to be removed from $RX_{2}{}^{\prime }$. Since $\Delta \omega_{1}$, $\theta_{1}$, $\Delta \omega_{2}$ and $\theta_{2}$ are estimated from the previous step, the interference signal in $RX2^{\prime }$ can be removed as

(21) $$RX_{2}{}^{\prime \prime }=RX_{2}{}^{\prime }-\varepsilon_{1}\times RX_{1}{}^{\prime }\times e^{-j\left[\theta_{2}-\theta_{1}+(\Delta \omega_{2}-\Delta \omega_{1}\right)t]}\;=a_{2}TX_{1}e^{-j\left[\theta_{2}-\theta_{1}+(\Delta \omega_{2}-\Delta \omega_{1}\right)t]}+b_{2}TX_{2}-\varepsilon_{1}\times a_{1}TX_{1}\times e^{-j\left[\theta_{2}-\theta_{1}+(\Delta \omega_{2}-\Delta \omega_{1}\right)t]}-\varepsilon_{1}\times b_{1}TX_{2}\;=\left(a_{2}-\varepsilon_{1}\times a_{1}\right)TX_{1}e^{-j\left[\theta_{2}-\theta_{1}+(\Delta \omega_{2}-\Delta \omega_{1}\right)t]}+\left(b_{2}-\varepsilon_{1}\times b_{1}\right)TX_{2},$$

where $\varepsilon_{1}$ is an adjustable coefficient that needs to be optimized to minimize the EVM. We do a search of $\varepsilon_{1}$ from its possible value to get the smallest EVM. Equation (19) indicates $a_{1}\gg b_{1}$, $a_{2}\ll b_{2}$. When the first component in (21) is removed, we have the smallest EVM. In this case,

(22) $$a_{2}-\varepsilon_{1}\times a_{1}=0$$

Then, (21) becomes

(23) $$RX_{2}{}^{\prime \prime }=\left(b_{2}-\varepsilon_{1}\times b_{1}\right)TX_{2}$$

Follow the same procedure to remove the interference signal in $RX_{1}{}^{\prime }$ and get

(24) $$RX_{1}{}^{\prime \prime }=RX_{1}{}^{\prime }-\varepsilon_{2}\times RX_{2}{}^{\prime }\times e^{j\left[\theta_{2}-\theta_{1}+(\Delta \omega_{2}-\Delta \omega_{1}\right)t]}=\left(a_{1}-\varepsilon_{2}a_{2}\right)TX_{1}$$

when

(25) $$b_{1}-\varepsilon_{2}\times b_{2}=0$$

**Figure 7:**
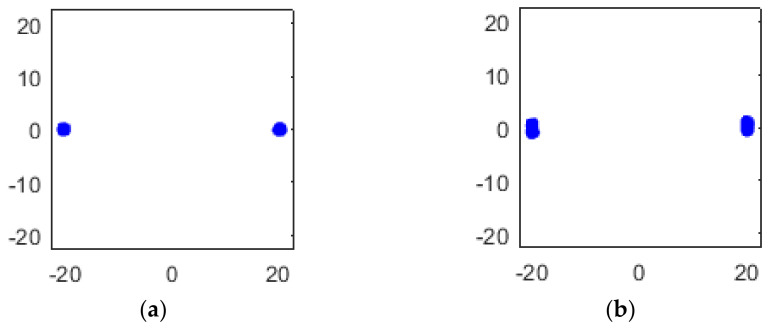
Simulated constellation diagrams of $RX_{1}{}^{\prime \prime }$ with $\Delta f_{2}-\Delta f_{1}$ of (**a**) 250 Hz and (**b**) 500 Hz.

The simulated constellation diagrams after equalization with different $\Delta \omega_{2}-\Delta \omega_{1}$ are shown in Figure 7. Compared with Figure 6, the constellation points are more concentrated.

### 5.4. Spread Spectrum Signature Code Selection

**Figure 8:**
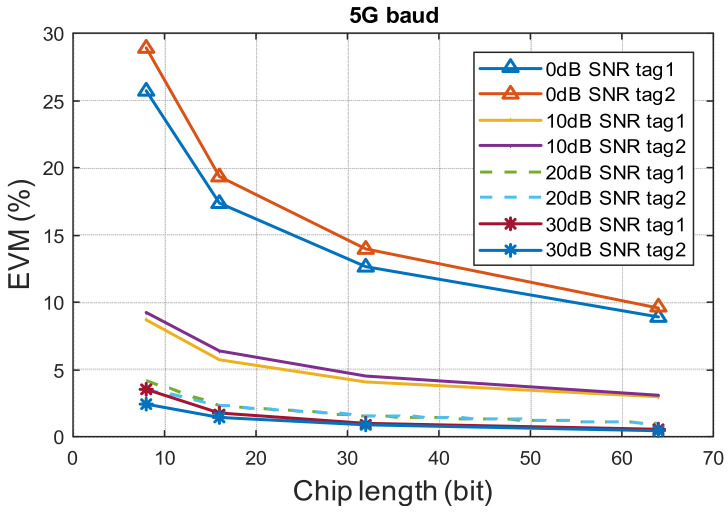
EVM VS. chip length with different SNR of 5Gbps chip rate signal in two tags scenario.

As introduced in section V-B, signature codes are used to separate signals from different tags. However, it is difficult to find a group of codes that guarantee zero cross-correlation with all different time offset Δt. In other words, (18) cannot be guaranteed to be zero with all possible Δt values. It is preferred to have $f\left(\Delta t\right)$ as small as possible compared to *L*. In this paper, the signature codes are a randomly generated set of pseudo-random sequences which have

(26) $$\underset{\Delta t\neq \;0}{\max}\left(f\left(\Delta t\right)\right)/L\approx 1/4$$

with different lengths from 8 bits to 64 bits. If the signal power from two tags is different due to distance, one signal’s power is less than 1/4 of the other one, then only one tag’s data can be recovered. Note that this ratio can be further reduced when a suitable signature code is selected. With this group of signature codes, increasing the length of the code doesn’t decrease the ratio between $\max\left(f\left(\Delta t\right)\right)$ and *L* significantly. However, increasing the length of the signature code chip can increase the SNR. As a result, the EVM reduces as the length of the signature code increases. A simulation result of the EVM of recovered signal from two tags with different chip lengths and SNR is shown in Figure 8. The chip rate is 5 Gbps. With the increase of chip length, higher signal power is obtained at the receiver side. As a result, the EVM decreases.

### 5.5. Radar Sensing Performance 

**Figure 9:**
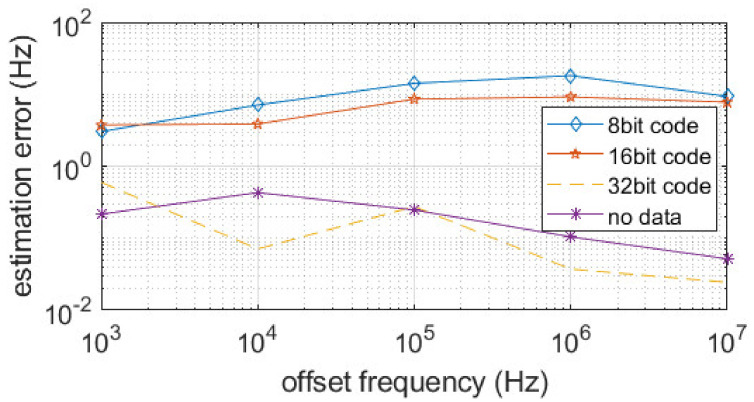
Frequency estimation performance with different lengths of signature code.

To study how modulated signals affect the radar sensing performance, we assume there is only one tag in the view. The radar sensing performance is related to the offset frequency estimation performance. We apply a two-step frequency estimation; First, apply FFT to get a coarse frequency estimation. Second, a phase-locked loop is applied to further track the frequency and phase offset. Here, we use a BPSK signal with a symbol rate of 5 Gbaud, and SNR of 20 dB. As Figure 9 shows, when the signature code has 8 or 16 bits, there is around a 10 Hz to 20 Hz estimation error. When the signature code has 32 bits, the estimation error is lower than 0.5 Hz, which is similar to no data modulation case. As a result, the SNR is the key factor in frequency estimation.

## 6. Tag Hardware and Test Result

**Figure 10:**
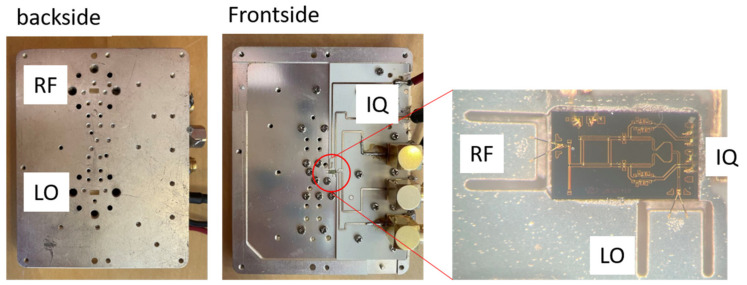
A picture of the packaged chip.

A package solution for the tag is presented in this section. A commercial E-band fundamental quadrature mixer MMIC (Gotmic gMR0012, Gothenburg, Sweden) [21] is packaged in a waveguide interfaced block as a tag in the proposed radar-communication system. The LO port is used as a tag input port, the RF port is used for modulated signal output, and baseband IQ ports are used to control theRF port phase-shifting regarding input LO signal. The photo of the packaged module is shown in Figure 10. On the back side of the module, two waveguide ports of WR-8 interfaces are used for signal input and output. 20 dBi waveguide interfaced antennas are connected to the tag during measurement. On the side of the module, differential quadrature IQ input port are provided by coaxial interfaces. On the front side of the module, a 200 um thick metal carrier board with etched slots are used for carrying MMIC and the U-shape slots are used for MMIC to waveguide transition. Also, a printed circuit board (PCB) is used for IQ signal connections to the MMIC.

**Figure 11:**
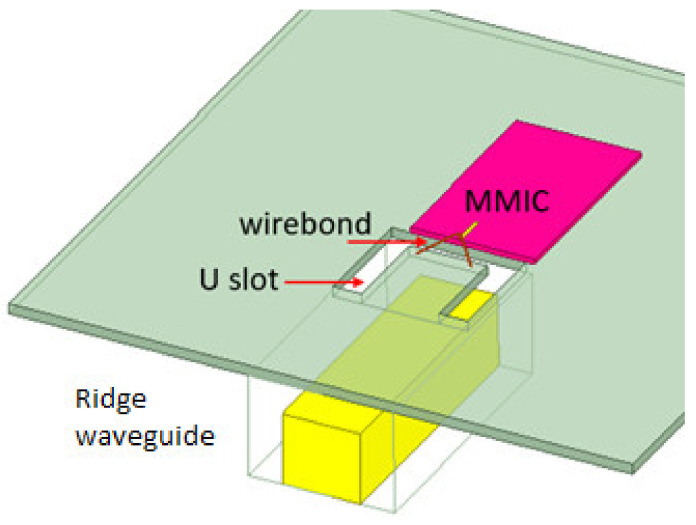
An illustration of the packaging concept.

**Figure 12:**
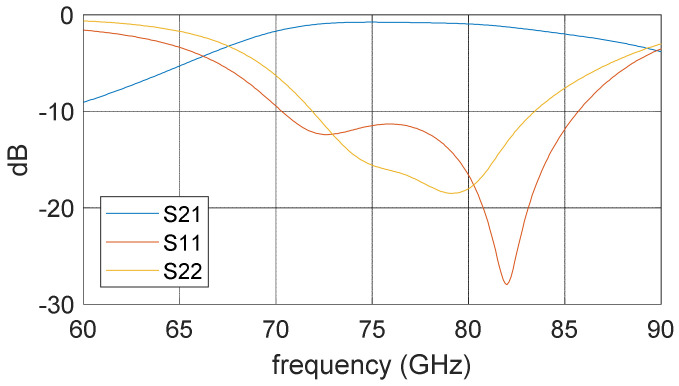
The S parameter measurement result of the transition including two bonding wires and a U-slot.

Different packaging solutions have been proposed, and many of them require a PCB. PCB introduces big loss at high frequencies, which is desired to be avoided in high frequency packaging solutions [22,23,24,25]. A novel substrate-less packaging solution is proposed in the design of the tag module. The LO and RF ports are directly wire bonded to the packaging block without using any dielectric substrate to reduce cost and avoid high electric loss at these frequencies. The packaging concept is illustrated in Figure 11. A ridge waveguide is used at the interface, whose upper wall is cut open with a U-shape slot. The upper wall (green part) is 200 um thick metal carrier which also holds MMIC on it. The ridge (yellow part) is manufactured by milling on a metal block (backside of the module, invisible in this figure). The MMIC is attached above this upper wall, and the signal PAD on the MMIC is connected to the upper wall with two bonding wires across the U-slot. These bond wires act as radiation probes, and the U-slot provides a coupling between the bond wire and the ridge waveguide. The length of this slot defines the coupling frequency. From the ridge waveguide to standard rectangular waveguide, a transition as described in [26] is used. This transition’s S parameter measurement result is presented in Figure 12, where port 1 is the lumped port on the MMIC and port 2 is the wave port at the ridge waveguide. The measurement exhibits insertion-loss less than 2 dB and return-loss less than −10 dB between 70–85 GHz.

**Figure 13:**
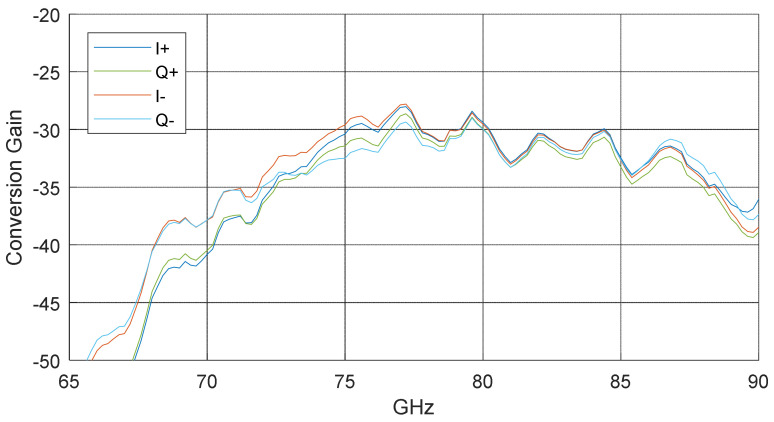
The measurement result of the conversion gain with different frequencies of the self-packaged tag.

The mixer used as a tag is a resistive mixer where the LO signal is split into quadrature paths, where different attenuation is applied on the path based on IQ input, and these paths are then combined into RF output. This operation principle implies an energy loss converting an LO signal to an RF signal. The packaged module is tested with −10 dBm LO input power and 1 GHz input at four different baseband ports (I+, I−, Q+, and Q−) independently. The conversion gain is plotted versus different LO frequencies in Figure 13. It can be seen that the mixer introduces a 35 dB conversion loss over the 70–90 GHz band.

## 7. Measurement Result and Discussion

Two measurements are demonstrated in this paper: the single tag measurement and the dual tag measurement. The single tag measurement shows the performance of the self-packaged tag with the LMF signal as a carrier. In the dual tag measurement, two tags are used to transmit two different data streams to simulate the multi-tag scenario.

### 7.1. Single Tag Test

**Figure 14:**
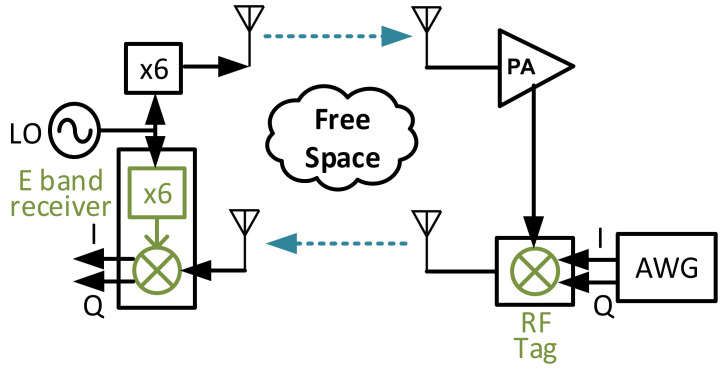
The measurement setup for a single tag test.

The measurement setup for the single tag test is shown in Figure 14. 

The LMF signal is generated from a signal generator (Agilent E8257D, Santa Clara, CA, USA) with a frequency between 12.533 GHz and 13.033 GHz. The chirp rate is 0.1 Hz. An E-band six times multiplier (AT Microwave AT-AM6-7186-12, Shanghai, China) is connected after the signal generator to generate the FMCW signal with a frequency from 75.2 GHz to 78.2 GHz. The FMCW signal is sent out through an antenna over the air. At the tag side, the FMCW is first amplified by a W-band power amplifier (QuinStar QPI-W01128, Torrance, CA, USA). An arbitrary wave generator (AWG, Keysight M8195A, Santa Rosa, CA, USA) is used to generate the baseband I/Q signal. The amplified FMCW signal is mixed with the baseband I/Q signal by the self-packaged RF tag. The RF signal is sent back to the radar side. On the radar side, an E-band receiver module (Gotmic gRSC0015, Gothenburg, Sweden) [27] is used to down-convert the RF signal. The module comprises a six times multiplier and a mixer. The FMCW signal is generated inside the receiver module by the multiplier and mixed with the received RF signal. The down-converted signal is sampled by an oscilloscope (Teledyne LabMater 10 Zi-A, Thousand Oaks, CA, USA) for further post-processing. The EVM and BER are estimated by the oscilloscope. The oscilloscope and the AWG are not synchronized and running freely. The measurement distance between antennas from the radar side to the tag side is around 65 cm.

**Figure 15:**
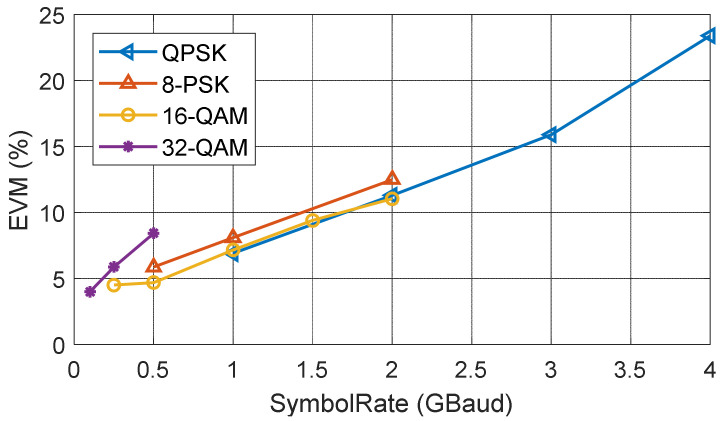
EVM VS. symbol rate with different modulation order signals of single tag measurement.

Signals with different modulation schemes have been transmitted over this backscatter communication system. The highest transmitted data rate is 8 Gbps with QPSK or 16-QAM. The highest tested modulation order signal is a 32-QAM signal with a data rate of 2.5 Gbps. Constellation diagrams of different modulation signals with their highest data rate are shown in Table 2. Their estimated EVM with different symbol rates are shown in Figure 15. Their BERs are all below 2 × 10^−4^. These measurement results show that the self-package tag is capable of transmitting a large bandwidth signal of 8 GHz and high modulation order signal of 32-QAM.

### 7.2. Two Tags Test

**Figure 16:**
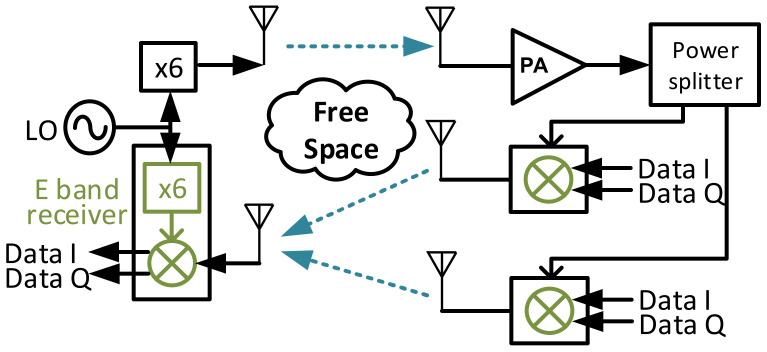
A measurement setup for the two tags test.

**Figure 17:**
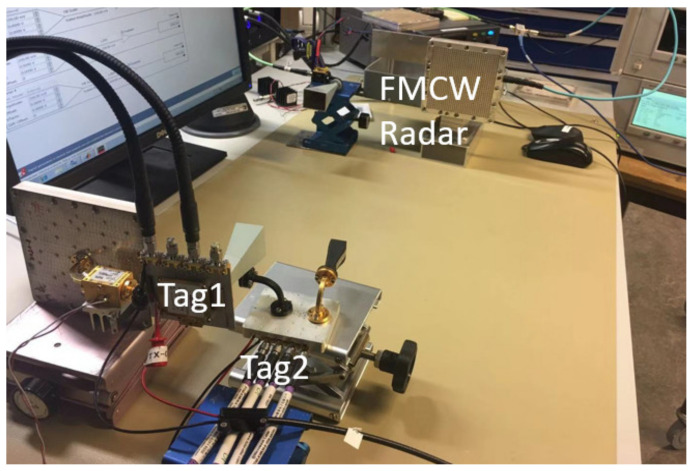
A photo of the measurement setup in the lab.

The measurement setup for the two tags test is shown in Figure 16. A photo of the lab setup is shown in Figure 17. Two tags and five antennas are used in the system. A power splitter is used on the tag side to provide the FMCW signal to both tags. In this case, the power of the FMCW signal provided to tags is 3 dB smaller than the single tag test. Note that the power splitter is not a necessary component in this system. It is used only due to the shortage of amplifiers. There should be an amplifier attached to each tag.

**Figure 18:**
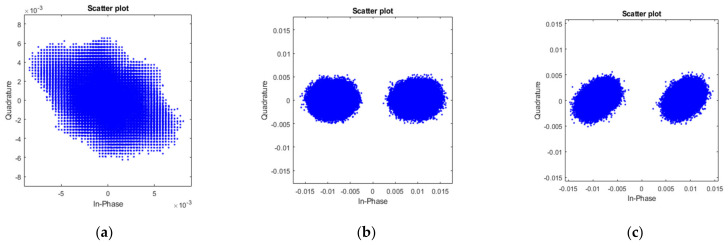
Constellation diagrams of (**a**) received signal from two tags with 625 Mbps BPSK of each tag, (**b**) demodulated signal from the first tag, and (**c**) demodulated signal from the second tag, where 8 bits signature code are used.

**Figure 19:**
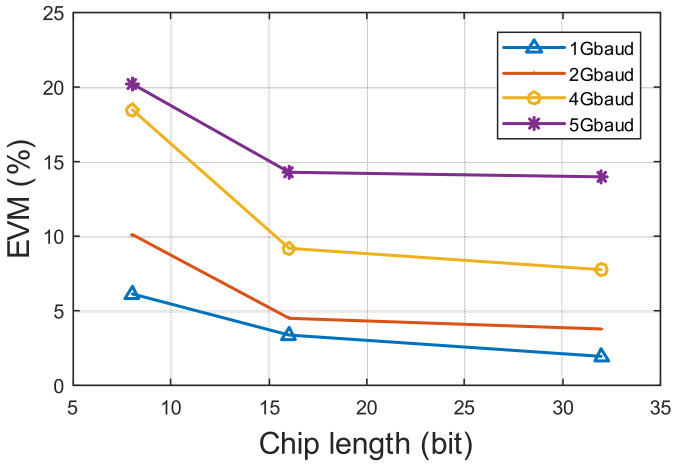
EVM VS. different chip length and different symbol rate of two-tag measurement.

The baseband signal for both tags is spectrum spread by using a different signature code. Different lengths of code and different symbol rates have been tested. In this case, only BPSK signals are transmitted. A 5 Gbps baseband signal has been transmitted with an 8-bit signature code so that the highest data rate that has been transmitted is 625 Mbps with both tags. The constellation diagrams of the received raw signal and the demodulated signals for two tags are shown in Figure 18. Before the demodulation, the received signal is a mixture of two signals from two tags. The constellation of it is cloudy. After applying the correlation with two signature codes, two signals from different tags are separated. Their constellations then become clear. By using different length signature codes, the EVM varies accordingly. The EVM of two signals are similar. Figure 19 shows the EVM decreases when chip length increases.

**Figure 20:**
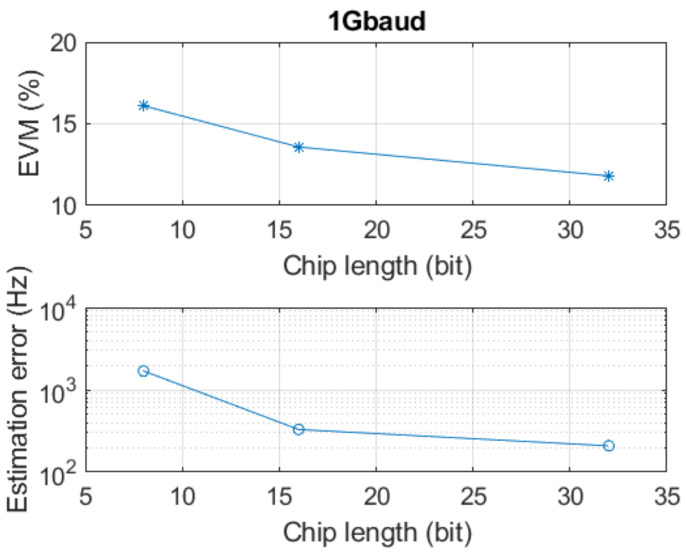
EVM and frequency estimation error with different chip length of two-tag measurement.

Due to the limited measurement distance in the lab and the slow sweep time of the signal synthesiser, the offset frequency at the receiver side is very small. To further test the system performance with larger offset frequencies, offset frequencies of 50 kHz and 49 kHz are added from the tag side by AWG. As Figure 20 shows, EVM and frequency estimation error decrease with chip length increase. Comparing Figure 19 and Figure 20, when using the same symbol rate signal in the system, higher offset frequencies cause higher EVM. For the 1 Gbuad signal used in the system, the EVM increase by around 10% when offset frequencies exist. If an FMCW with 1 GHz bandwidth and 1 ms duration time is used in this system, the frequency estimation error shown in Figure 20 will cause a 60 mm to 500 mm range estimation error with different chip lengths. This is due to an immature frequency estimation solution, and it can be further improved with more advanced processing methods.

### 7.3. Link Budget

The six times multiplier on the radar side gives an output power of 10 dBm. The antenna connected to it is a horn antenna (AINFO LB-12-25-A, Chengdu, China) that produces a 25 dBi gain. At the tag side, another slot array antenna provides another 22 dBi gain. The distance between the tag and the radar is around 65 cm, which introduces around 66.4 dB of free space path loss. Thus, the received LO signal at the tag side in our case is around −9.4 dBm. After using an amplifier (QuinStar QPI-W01128, Torrance, CA, USA) and a 3 dB power splitter, the LO power used for the tag is around 8 dBm without considering cable loss.

A pair of I and Q channel is used for each tag with an input power of −10 dBm at each channel. The conversion loss is around 30 dB at the frequency that we used. The RF output power from the tag is around −37 dBm. Antennas in use for two tags have 25 dBi and 20 dBi gain, respectively. On the radar side, the slot array antenna provides 22 dBi gain. The received signal power at the radar side from each tag is −56.4 dBm and −61.4 dBm, respectively.

## 8. Conclusions

The proposed multi-channel backscatter communication with FMCW radar is tested with commercial E-band frontend modules at 75.2 to 78.2 GHz. A novel package solution for the RF tag is proposed. The tag performance is tested for different modulation schemes and different data rates. For the single tag measurement, it demonstrates the highest data rate of 8 Gbps and the highest modulation order signal of 32-QAM compared to other related works. The frequency offset between the transmitter and the receiver can be used for range measurement. Its accuracy is related to the SNR and chip length of signature codes in use. For the multi-tag measurement, signature codes are used to separate signals from two tags. Blind equalization is applied to further decrease the EVM. By using signature codes, tags can be freely deployed.

## Figures and Tables

**Table 2:** Constellation diagrams of different modulation order signals with their highest achieved data rate of single tag measurement.

Constellation	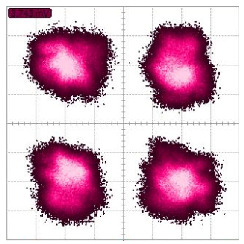	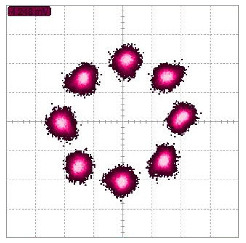	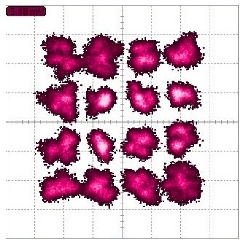	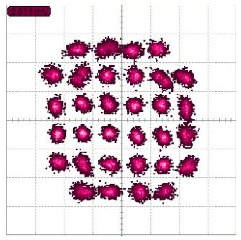
Modulation	QPSK	8-PSK	16-QAM	32-QAM
Symbol rate	4 Gbaud	2 Gbaud	2 Gbaud	500 Mbaud
Bit rate	8 Gbps	6 Gbps	8 Gbps	2.5 Gbps
Bit error rate	9.9 × 10^−6^	1.5 × 10^−4^	1.93 × 10^−5^	1.3 × 10^−4^
EVM	23.44%	12.51%	11.04%	8.43%
